# Plastination with low viscosity silicone: strategy for less tissue shrinkage

**DOI:** 10.1590/1414-431X2022e11962

**Published:** 2022-07-13

**Authors:** Y.F. Monteiro, M.V.F. Silva, A.P.S.V. Bittencourt, A.S. Bittencourt

**Affiliations:** 1 Laboratório de Plastinação, Departamento de Morfologia, Centro de Ciências da Saúde, Universidade Federal do Espírito Santo, Vitória, ES, Brasil; 2 Programa de Pós-Graduação em Bioquímica, Universidade Federal do Espírito Santo, Vitória, ES, Brasil; 3 Departamento de Ciências Fisiológicas, Universidade Federal do Espírito Santo, Vitória, ES, Brasil

**Keywords:** Shrinkage, Plastination, Silicone, Preservation, Viscosity

## Abstract

Plastination is an anatomical technique for preserving biological tissues based on the principle of replacing body fluids with a curable polymer. An inconvenient aspect of this technique is the tissue shrinkage it causes; several studies seek ways to reduce or avoid this shrinkage. Additionally, there are no studies in the literature that quantitatively evaluate the use of low viscosity silicones in plastination having shrinkage of tissue as a parameter. Therefore, this study aimed to evaluate the use of Silicones S10 (Biodur) and P1 (Polisil) in the plastination of different types of biological tissues of a sliced human body, having as a parameter the tissue shrinkage caused in the forced impregnation stage. Human cardiac, pulmonary, splenic, renal, hepatic, muscular, and bone tissues were analyzed. For such purpose, a male human body was used, sliced in 13-15-mm-thick pieces, having as a parameter the before and the after plastination with the different silicones. The standard protocol of the plastination of the slices was followed: dehydration, forced impregnation, and curation. Half of the pieces obtained were plastinated with silicone P1 (group P1) and the other half with S10 (group S10). All tissues and anatomical segments analyzed in this study showed less or equal shrinkage when plastination of the control group (S10) was compared with that of the P1 group. Therefore, we concluded that the lower viscosity silicone promoted less tissue shrinkage, making it a viable alternative to the reference.

## Introduction

Plastination is a technique for the preservation of anatomical specimens developed in 1977 by the German physician and professor Gunther von Hagens, in which biological tissue is kept inert, preserved, realistic, and antiseptic for an indefinite amount of time (1). Moreover, the technique avoids the use of toxic preservative solutions for the maintenance of anatomical specimens, such as formaldehyde, and greatly increases the durability of the parts, allowing their handling.

The principle of this conservation method is the substitution of tissue fluids by a curable polymer; this is achieved, according to von Hagens et al. (1), through a process consisting basically of four fundamental steps: formalin fixation, acetone dehydration, forced impregnation with the polymer of choice, and chemical or luminous catalysis of this polymer.

Although plastination was created approximately 40 years ago, it is only in the last decade that it became widespread and gained prominence (2). This, along with the development of new technologies, has generated a fertile field for scientific research related to this technique. Thus, many related studies have emerged in the most diverse areas of knowledge, such as medicine, chemistry, biochemistry, histology, human and veterinary anatomy, education, embryology, pathology, and others (3).

The main polymers used are epoxy, polyester, and silicone, the latter being the most used due to its wide range of possibilities: from fragments of biological tissue to large animals (4,5).

The silicone of reference, used worldwide in the plastination technique, is the S10 of the German brand Biodur^®^ (Germany), which has been specially developed and tested for this purpose (4). However, many plastinators have been testing the use of national silicones. These experiments aim to bypass the need to import products and the inherent bureaucracy, reduce supply expenses, and explore alternative polymers for use in plastination.

One of the questions raised in relation to the plastination of biological tissues is regarding the shrinkage inherent to the technique, which occurs mainly during the forced impregnation stage, when acetone is replaced by the polymer (4,5). Since it is one of the drawbacks of the technique, several studies seek ways to avoid and reduce the tissue shrinkage that occurs in plastination. The literature, however, lacks an in-depth study investigating the use of silicones with different viscosities and their effects on the shrinkage rate in different types of biological tissues. In this regard, research that tests different polymers can be of great benefit to the technique. Furthermore, research into alternatives to the reference silicones (Biodur^®^) would provide important advantages in terms of acquisition cost.

Thus, the objective of this research was to evaluate the use of a low viscosity silicone (Poliplast 1 - P1; Polisil^®^, Brazil) and the reference silicone (S10, Biodur^®^) in the plastination of different types of biological tissues of a sliced human body, having tissue shrinkage as the parameter.

## Material and Methods

Aiming to facilitate description, the research was divided into two parts: the plastination of the sliced body and the evaluation of tissue shrinkage.

### Sliced body plastination

The human body used in the research was part of the collection of the Anatomy Sector of the Department of Morphology, located in the Center of Health Sciences of the Federal University of Espírito Santo (UFES, Brazil). All the documentation of the incoming unclaimed body was dutifully regularized, in accordance with Federal Law No. 8,501 (November 30, 1992) that authorizes its use for teaching and research purposes. The chosen body was of a man, aged between 60 and 65 years, approximately 1.65 meters tall. The body had already been fixed and preserved in 10% formalin for approximately 5 years.

First, the body was frozen in an anatomical position in a horizontal freezer, at -25°C for 48 h. The feet were then cut at ankle level, the hands at wrist level, and the head was cut with the neck. Hands and feet were not considered in this study due to the lack of standardization of the thickness and cut plans of the pieces. As for the head, the segment would need to undergo special shock-freezing procedures for slicing the central nervous system (6,7), which was not performed. The left leg was also not used in the research, since an intramedullary rod in the tibia bone impeded slicing. Moreover, the nervous tissue did not present good fixation quality for analysis. The body was, then, embedded in polyurethane resin (PU) and transferred to a horizontal freezer for another seven days at -25°C. The embedding and freezing facilitated the subsequent slicing in an aligned manner, reducing the risk of losing the cutting plane.

The slicing was carried out with the aid of a Skymsen SSI No. 1974 band saw (Brazil) in the transverse plane of the body, with slices between 13 and 15 millimeters thick.

After this step, all slices were labeled and identified in ascending numerical order, starting with 01 for the upper chest. In addition to the number, the upper and lower limbs were identified with the letters D for right (*direita*) or E for left (*esquerda*).

**Figure 1:**
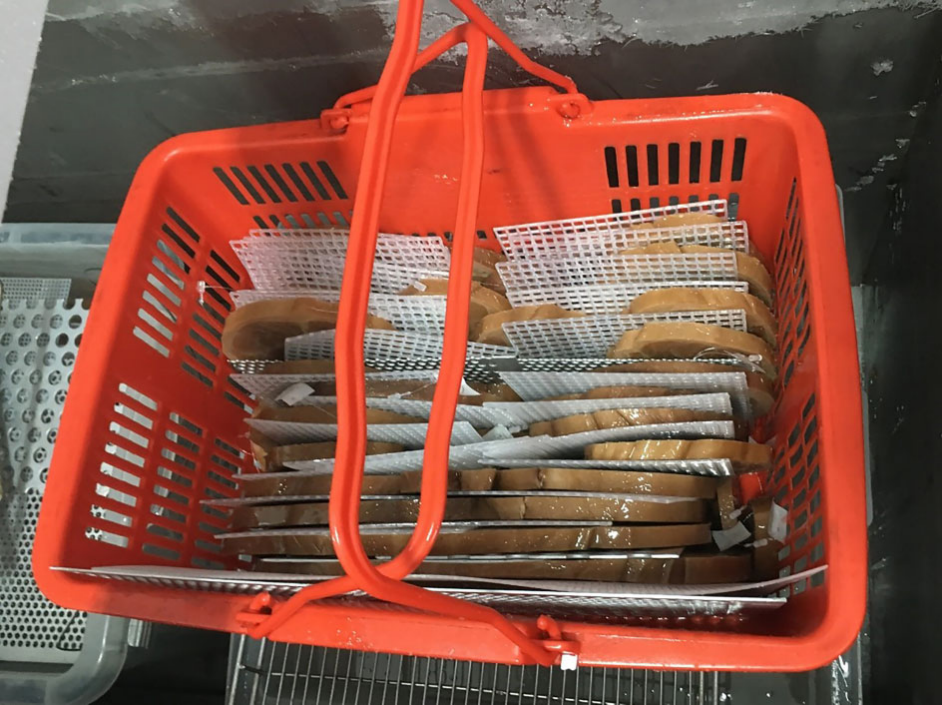
Draining step of slices inside the vacuum chamber.

The plastination technique was performed according to the protocol proposed by von Hagens et al. (1), divided into 4 main stages: fixation, dehydration, forced impregnation, and curation/chemical catalysis. Fixation had already been performed previously, using 10% formalin solution. After slicing, as already described, the slices were arranged in a vertical position in baskets and separated by perforated plastic sheets. This arrangement ensures that all cuts would have the same impregnation conditions, without superimposing, avoiding direct contact, and creating spaces between slices for a good dehydration and forced impregnation. Then, dehydration occurred at low temperature (-25°C) with 4 weekly acetone immersions of concentrations 95, 95, 100, and 100% (v/v) consecutively in a freezer. The dehydration stage with acetone was considered complete when a purity greater than 99% (v/v) was reached. At this point, the pieces were separated into a control group (S10) and a test group (P1). The control group consisted of the pieces identified by an even number, totaling 77 slices, and the test group was composed of pieces identified by odd numbers, totaling 81 slices. From then on, inside the vacuum chamber (dimensions: 60×50×110 cm), the pieces were immersed in the reactive mixture of cold impregnation (-18°C) composed of the silicone to be tested (S10 or P1) and its respective dibutyltin dilaurate (DBTDL) catalyst in the proportion of 100:1 (m/m) for 24 h. Vacuum was then applied slowly and progressively; the bubble/second pattern at the same observation point served as a parameter for vacuum adjustment (8). For standardization of impregnation, all the body slices were impregnated at the same time. Vacuum progression was measured with a digital and a mercury manometer. When the bubbles ceased to appear on the silicone surface and the maximum vacuum was reached by the pump, the stage was considered complete, lasting 26 days and reaching the minimum pressure (maximum vacuum) of 8 mmHg. Then, after turning off the pump and restoring the atmospheric pressure inside the vacuum chamber, the slices remained in silicone for an additional 24 h (8). Thus, the slices were suspended in the vacuum chamber (-15°C) for 48 h, followed by 5 days at room temperature (20-25°C) for drainage of excess silicone (Figure 1). This time was necessary for efficient drainage and to prevent the silicone from leaking and polymerizing during the curing step to avoid a shiny and artificial appearance.

In the chemical cure, the crosslink agent tetraethyl orthosilicate (TEOS) was vaporized in a closed bag containing the arranged parts undergoing silicone hardening. All slices were equally and simultaneously drained and cured. After two days of curing, the specimens were ready. All plastination of the material was performed in the Plastination Laboratory of the UFES.

### Shrinkage evaluation of the slices and of the different biological tissues

Since the best way to quantify the level of tissue shrinkage of plastinated specimens is by volume difference (9), the volumes (mL) of each slice were measured both before impregnation and after chemical curing. This was done to verify the influence of silicones with different viscosities on the tissue shrinkage caused by the forced impregnation step with the polymer. For this assessment, the pieces were submerged in glass basins filled with pure acetone at room temperature (20-25°C), and the volume of displaced liquid was recorded to determine the volume of the piece. Since the pieces varied in sizes, four previously calibrated glass basins of different sizes were used. For standardization, the same glassware and basins were used in both steps of volume measurement for the same pieces. The volumetric shrinkage of each piece is reported as a percentage and was calculated according to Equation 1:

$$\frac{\text{volume}\left(\text{mL}\right)\text{before impregnation}–\text{volume}\left(\text{mL}\right)\text{after curation}}{\text{volume}\left(\text{mL}\right)\text{before impregnation}\times \;100\;}=\text{\% shrinkage}$$ (Eq. 1)

The overall mean (SD) shrinkage was estimated for each piece and by anatomical segment. The volumes were measured after chemical cure, since measurement of the rigid pieces is easier than with liquid silicone. Also, the use of liquid to measure the volume before curing could interfere with the curing step. Volume measurements were taken with the same glass basins used before impregnation filled with water at room temperature (20-25°C). According to the value reported in the polymer technical data sheets, the shrinkage caused by the cure is uniform and less than 0.5%, that is, standardized for all cuts.

**Figure 2:**
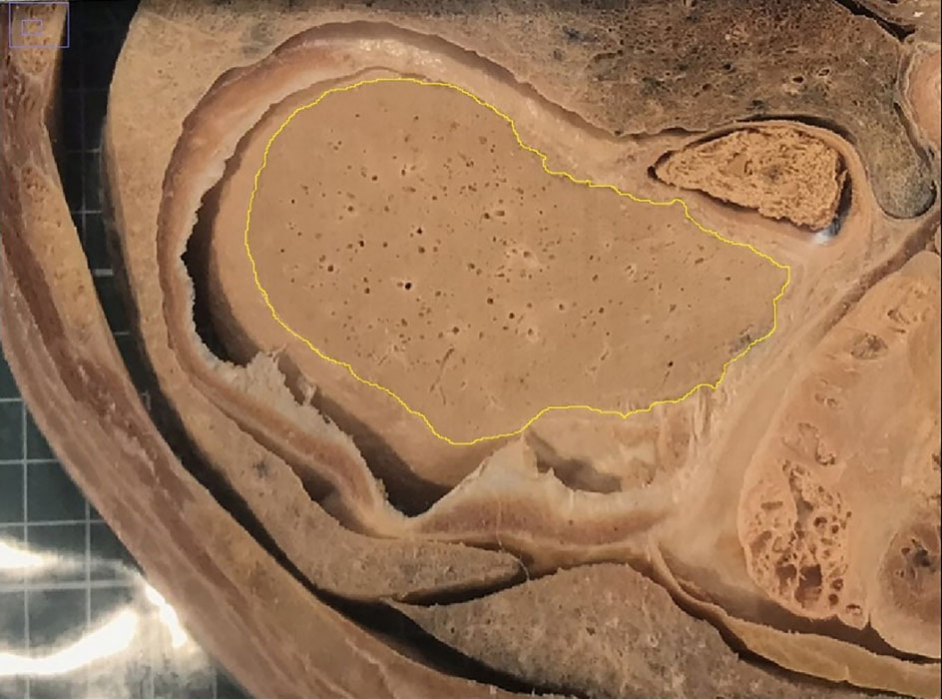
Measurement of the area (cm^2^) of the liver on the upper side of the slice before forced impregnation by the ImageJ program. The yellow demarcation and the measuring scale (one centimeter) is reported in the software for calculation.

The shrinkage of the different types of tissues was not possible to measure, since organ segments were fixed in different slices. Thus, the area (cm^2^) of the tissues of interest, within its slice, was used as a parameter for the shrinkage measurement. The analyzed tissues were: cardiac, pulmonary, hepatic, splenic, renal, muscle, skeletal muscle, and bone. For the purpose of estimating the shrinkage of muscle tissue, the areas of the gracilis, sartorius, and the rectus femoris muscles were measured and for the evaluation of bone tissue, the humerus and femur bones were considered. These bones and muscles were chosen due to the easy demarcation of their boundaries in the slices and the large limbs in which they are located compared with other muscles, generating a larger number of samples. To standardize the analysis, all pieces were photographed immediately before impregnation and after chemical curing, under the same conditions for both moments: at the same distance and angulation between the camera and the piece, which was positioned on a tray with a measurement scale. The area of the tissues was calculated with the ImageJ software, measuring the total surface area of the upper side of the organ slice, as shown in Figure 2. The software estimates the area from the pixel count of the photos, having as parameter an informed scale. From the measurements, the shrinkage percentage per area of analyzed tissue was estimated using Equation 2:

$$\frac{\text{area}\left({\text{cm}}^{2}\right)\text{before impregnation}–\text{area}\left({\text{cm}}^{2}\right)\text{after curation}}{\text{area}\left({\text{cm}}^{2}\right)\text{before impregnation}\times \;100\;}=\text{\% shrinkage}$$ (Eq. 2)

The mean ± standard deviation (SD) shrinkage of the tissues was also estimated.

The Bartlett test with 5% significance was used to analyze the homogeneity of the variance of all data sets. If positive, one-way ANOVA was used to indicate possible differences in segment or tissue shrinkage between groups, with a 5% significance level; if negative, a Kruskal-Wallis test was performed. Tukey's test was performed, with a 95% confidence interval, for the analysis of the upper and lower limits of the data and mean comparisons. Additionally, a one-way ANOVA, with a 5% significance level, was performed to compare the mean shrinkage of tissue and segment within the same experimental groups (S10 or P1). All statistical analyses were performed with the Statistical Package for the Social Sciences (SPSS for Windows 8; IBM, USA), Microsoft Excel 2010 (Microsoft Office System 2010, USA), and R (R 2020) programs (R Core Team).

## Results and Discussion

In this study, 158 human slices were plastinated and considered for the analysis of different anatomical segments: 15 of the thorax, 20 of the abdomen, 9 of the pelvis, 63 of the lower limb (LL), and 51 of the upper limb (UL).

Table 1 shows the mean percent (±SD) of each anatomical segment estimated from the initial and final volumes. The table also shows the value, in %, of the minimum and maximum shrinkage of the slices for each anatomical segment group. The minimum and maximum values for all slices of the group are also shown, with the mean calculated from the total number of samples in this group.

**Table 1:** Maximum, minimum, and mean±SD percent shrinkage in pieces according to anatomical segments and silicones used (S10 or P1).

Silicone/Segment	No. of slices	Shrinkage (%)		
		Minimum	Maximum	Mean±SD
S10				
Thorax	7	11.7	15.6	13.8±1.2
Abdomen	10	12.1	21.3	17.7±3.4
Pelvis	5	11.4	17.7	14.1±2.3
Upper limb	25	15.4	28.5	23.0±4.3
Lower limb	30	9.5	26.6	19.9±5.1
All	77	9.5	28.5	19.6±5.2
P1				
Thorax	8	7.3	14.8	11.3±2.3
Abdomen	10	8	15.9	13.2±3.4
Pelvis	4	5.7	13.6	8.7±3.8
Upper limb	26	3.3	12.7	6.1±3.6
Lower limb	33	1.5	18.2	9.7±4.9
All	81	1.5	18.2	9.1±4.6

All segments showed a significant difference in mean shrinkage between silicone groups (ANOVA).

The anatomical segment with the lowest shrinkage was the upper limb (6.1±3.6%) for the P1 silicone and thorax (13.8±1.2%) for the S10 silicone. The one with the greatest shrinkage was the abdomen (13.2±3.4%) for P1 and upper limb (23.0±4.3%) for S10.

**Figure 3:**
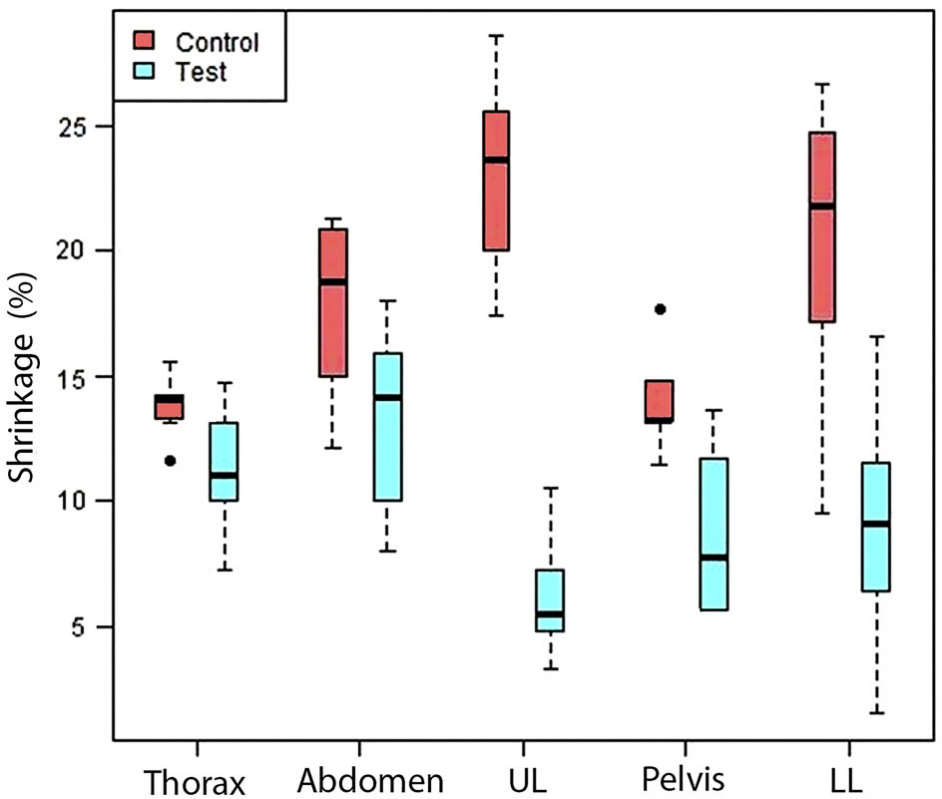
Comparison of percent volumetric shrinkage by anatomical segment. Control: S10 silicone; Test: P1 silicone. Data are reported as median and interquartile range. Between-group differences were all significant (P<0.05; Kruskal-Wallis test). UL: upper limb; LL: lower limb.

Figure 3 shows the comparison of segment shrinkage variations for the S10 and P1 silicone groups, in which the distribution and symmetry of the data are shown with the minimum and maximum values of retraction for each type of tissue.

Table 1 and Figure 3 show that certain segments present very different minimum and maximum shrinkage percent within the same silicone group. This difference was also observed when we compared different segments in the same group. These differences occurred because a variety of factors that influence the level of shrinkage, such as the types of tissues in the pieces, proportion of bone tissue/soft tissues, and contact surface. Other studies have already shown that different biological tissues react differently in the impregnation stage, affecting the degrees of shrinkage (4,5). Pieces with a greater amount of adipose tissue, for example, retract more (4). Of all body tissues, the bone suffers less shrinkage in the plastination technique, since its constitution is mostly inorganic matter (65-75%) - mostly in the form of hydroxyapatite crystals - making it a very rigid tissue, with almost no shrinkage (10,11). Thus, pieces that have a large proportion of bone tissue generally shrink less. For example, in the lower limb segment, the slices at knee level have a high bone tissue-to-soft tissue rate, causing these pieces to shrink less than those at thigh level. Another important factor that influences the rate of shrinkage is the area of contact between the dehydrated tissue and the surrounding silicone. The larger the area of contact of the segment or piece, the more efficient the acetone/silicone exchange. Therefore, pieces that have many recesses, such as those with intestine, tend to be impregnated more easily and shrink less. These factors apply to tissues in the same segment and in different anatomical segments.

The main evaluation carried out in this work to achieve the proposed objective was the comparison of segment and tissue retractions between groups P1 and S10, but although this did not yield very clear contributions, a comparison between segments of the same group was also carried out.

The upper limb was the anatomical segment that had the greatest proportional difference in retraction comparing the experimental groups (S10 and P1), since it retracted approximately 3.8 times more with S10 than with P1 (Table 1 and Figure 3). The reason for such discrepancy is not clear, as several factors can influence the final result of the process, such as the non-linear physicochemical behavior of silicone, which can vary depending on its viscosity and interaction with tissues of different biochemical constitutions. Corroborating this hypothesis, the study by Monteiro et al. (12) shows that silicones with different viscosities behave in a non-proportional way with the change in temperature.

We also estimated the percent area shrinkage of organs (tissues), as shown in Table 2. To estimate the mean general shrinkage of bilateral organs, such as the lungs, the shrinkage values of the right and left structures were used. To estimate the mean shrinkage of all tissues, the values measured in the biological tissue samples were used separately, including right and left sides, when applicable.

**Table 2:** Mean percent shrinkage (MPS) ±SD per organ/tissue (area measurement).

Anatomical structure	MPS S10 (%)	No. of samples	MPS P1 (%)	No. of Samples
All tissues*	15.1±10.6	102	7±5	105
Heart	10.2±8	2	2.7±1.7	2
Left lung*	13.1±3.7	6	5.5±1.9	6
Right lung*	12.3±7.7	6	4.4±2.9	6
Lung: general*	12.7±5.8	12	4.9±2.5	12
Right kidney	19.6±4.1	3	12.5±4.9	3
Left kidney	16.7±1.8	3	16.6±0.3	3
Kidney: general	18.2±3.2	6	14.5±3.8	6
Liver*	14.7±5.1	4	4.2±0.8	4
Spleen*	20.3±7.6	3	9.8±1	3
Humerus	1.1±0.8	12	1.9±1.3	10
Femur	1.3±0.6	17	1.7±1.1	18
Bones: general*	1.2±0.7	29	1.8±1.2	28
Rectus femoris*	25.1±6.3	12	8.1±4.3	14
Sartorius*	23.9±5.3	17	11.2±3.3	18
Gracilis*	23.2±4.6	17	9.7±3.6	18
Muscles: general*	23.9±5.2	46	9.8±3.8	50

* P<0.05 between groups (S10 and P1) (ANOVA).

The tissue with the lowest shrinkage rate in plastination, both with P1 and S10 silicone, was bone, as expected, with 1.8±1.2% and 1.2±0.7%, respectively. The tissue with the highest mean shrinkage percentage with P1 was renal (14.5 ±3.8%), and with S10 was muscular (23.9±5.2%).

**Figure 4:**
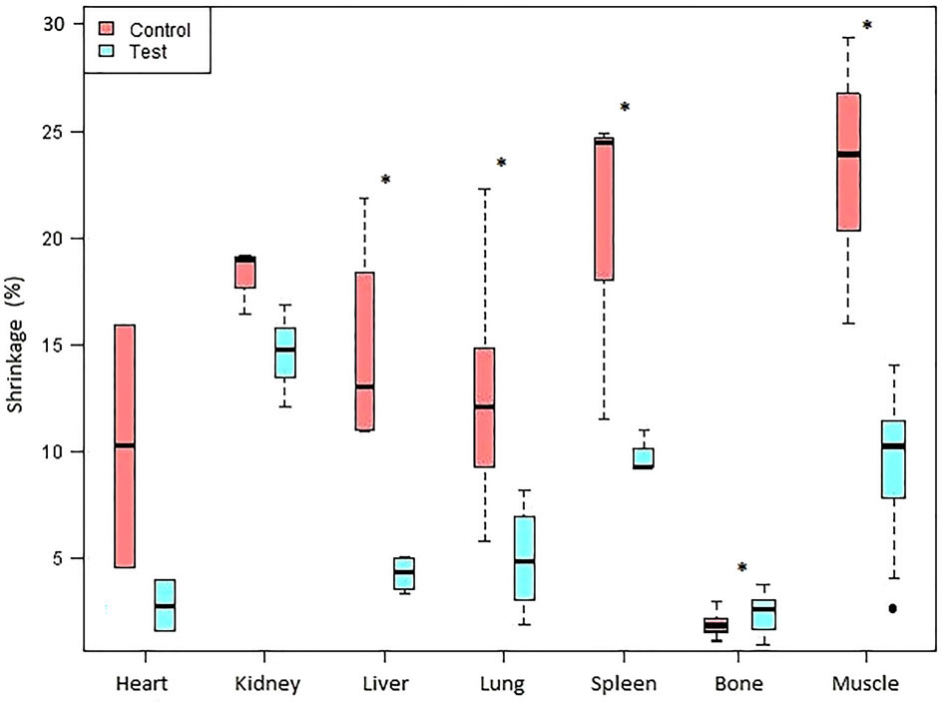
Comparison of percent shrinkage by biological tissues in the control (S10) and test (P1) groups. Data are reported as median and interquartile range. *P<0.05 between groups (S10 and P1). (P<0.05; Kruskal-Wallis test).

Figure 4 shows the comparisons of tissue shrinkage for the S10 and P1 silicone groups, with the distribution and symmetry of the data and minimum and maximum values of retraction for each type of tissue.

Muscle had the highest mean shrinkage value for silicone S10, probably because of the contractile fibers that are resilient rather than rigid when fixed in formalin, compared to the other analyzed tissues. Up to 92% of the total volume of muscle tissue is muscle fibers, leaving a small percentage for more rigid structural collagen fibers, which is a necessary condition for the contractile function. In addition, the loose connective tissue that fills the spaces between muscle cells has little resistance to retraction (13). This degree of shrinkage corroborates the results for shrinkage of the lower limbs and upper limbs, since these are, on average, the segments with the highest proportion of muscle tissue and showed the greatest shrinkage among the analyzed segments (Table 2 and Figure 4).

In their work, Guimarães et al. (14) presented a scale of mechanical stiffness of different fresh biological tissues, an important factor that may be related to retraction. The authors also cited several factors that modify biomechanical rigidity, such as age, presence of disease, and lifestyle. On the presented scale, among other organs, kidney, liver, lung, and bones are in order of increasing stiffness. Thus, the absolute values of shrinkage found in our research (Table 2) followed the same order as this scale (14). We assume that more malleable tissues have a greater tendency to present greater tissue retraction in the forced impregnation stage, since the rigidity of the tissue counteracts the shrinkage. In the fixation stage, formalin alters the mechanical properties, increasing the rigidity of biological tissues, mainly through synthesis of cross-bridges in collagen proteins (15). However, based on our results and the work mentioned above, it seems that the proportionality of the stiffness of the different tissues is maintained after fixation.

Starchik and Henry (5) also measured the degree of volumetric shrinkage of some biological tissues evaluated in the present study, including kidney, liver, and heart. They noted that kidney tissue had the greatest shrinkage, followed by liver and heart tissue. Although no statistical difference between silicones was observed for the same tissues, perhaps because of the small sample size, the absolute values obtained for the tested silicones (kidney > liver > cardiac) are consistent with Starchik and Henry’s finding (Figure 4).

Different tissues have different shrinkage rates in the forced impregnation stage within the same silicone group (Table 2). Among the main factors responsible for this are the biochemical composition of tissues, contact surface, and extracellular structure. Tissue composition is an important factor for shrinkage, since a greater amount of tissue water usually causes a greater shrinkage because the water is replaced by acetone and later by the polymer. In addition to water, part of the lipids is also removed from the tissues in dehydration, since they are solubilized in the solvent used, acetone (16), which is later also replaced by the polymer. In the impregnation stage, the contact surface, as explained earlier, allows a larger area of exchange between acetone in the tissue and the polymer around it. The extracellular structure or matrix is different for each tissue (with more or less liquid content), with structural proteins and macromolecules in general (9), affecting tissue dehydration and stiffness, i.e., the susceptibility to greater or lesser shrinkage. For a better understanding of the reasons for the different shrinkage rates of segments and tissues found in this study, a microscopic analysis of the tissues before and after forced impregnation would be extremely useful.

The homogeneity of variance, one of the premises for ANOVA, was analyzed by Bartlett tests for all tissues and segments plastinated with the different silicones. Of these, only three sets (all tissues, liver, and spleen) presented P-values less than 0.05 and, therefore, violated the premise of homogeneity. Thus, one-way ANOVA was performed for the analysis of variance for all data sets - except for the three previously mentioned sets, which were analyzed with Kruskal-Wallis test, a non-parametric variation of ANOVA (Table 3). The lower and upper limits were computed using the Tukey's test.

**Table 3:** Tissue or segment shrinkage values tested with P1 and S10 silicones.

Groups	Variance analysis		Tukey	
	F or χ^2^	P-value	Lwr	Upr
All tissues*	15.67	**7.541** ^-5^	-	-
Bones: general	4,507	**0.043**	0.020	1.148
Femur	1,876	0.190	-0.244	1.136
Humerus	2,359	0.159	-0.385	2.016
Muscles: general	220,640	**0.000**	-16.067	-12.234
Gracilis	113,530	**0.000**	-16.208	-10.829
Rectus femoris	77,660	**0.000**	-22.047	-13.235
Sartorius	55,645	**0.000**	-15.686	-8.744
Organs: general	21,229	**0.000**	-11.674	-4.528
Heart	1,668	0.326	-32.421	17.441
Kidney	4,896	0.091	-8.183	0.925
Liver*	5,333	**0.02092**	-	-
Lung	9,787	**0.011**	-13.334	-2.241
Spleen*	3,857	**0.04953**	-	-
Segments: General	132,490	**0.000**	-11.225	-7.928
Thorax	6,109	**0.028**	-4.593	-0.309
Abdomen	8,467	**0.009**	-7.664	-1.237
Upper limb	203,630	**0.000**	-19.132	-14.297
Pelvis	6,835	**0.035**	-10.254	-0.514
Lower limb	70,602	**0.000**	-13.725	-8.409

F values for ANOVA. *Kruskal-Wallis test or χ^2^ values. Lwr and Upr: 95% lower and upper limits of the Tukey test.

The Tukey’s test was used to assess the differences between means of the different groups and was used as a complement to the ANOVA results. If the range between the lower and upper limits included zero, there was no significant difference between the groups. As seen in Table 3, the range of all subgroups of segments and biological tissues that showed significance in the comparison between silicones did not include the zero value, confirming a significant difference between means.

Table 3 shows that the difference in shrinkage caused by plastination in both silicone groups (P1 and S10) was significant for all segments and tissues (P<0.05), except for the sets of femoral bone, humerus bone, heart, and kidney. As expected and already discussed, the shrinkage in bone tissue is negligible and served as a standard for validation of the shrinkage analysis method by the difference in area. Regarding the cardiac tissue group, the P-value was not significant probably due to the small number of samples (n=2 for each group) added to the large standard deviation. Although not significant, the analysis of the results of the cardiac and renal tissue groups also showed lower mean shrinkage with the P1 silicone, suggesting a tendency for significance. Larger samples would probably yield a significant difference in shrinkage with P1 in all subgroups tested.

Thus, the P1 silicone induced significantly less shrinkage in general, as well as in the different types of tissues and segments.

The smaller shrinkage of tissues and segments in the P1 silicone group was mainly due to its lower viscosity. The P1 silicone has viscosity estimated in 420 mPa/s, whereas the viscosity of S10 silicone is possibly 1250 mPa/s, both estimated at the impregnation temperature used in this study (-18°C) (11).

The higher the viscosity of a silicone, the greater the shrinkage of biological tissues in the impregnation stage (5). This is mainly due to the fact that the greater the viscosity of the silicone, the greater the resistance to its permeation in the tissue in the impregnation stage, when acetone is volatized faster (leaving the tissue more easily) and the silicone penetrates/flows at a slower rate (the more viscous, the lower the rate), causing tissues to shrink. Therefore, the use of low viscosity silicones may be preferable when seeking a lower final tissue shrinkage in the specimen.

**Figure 5:**
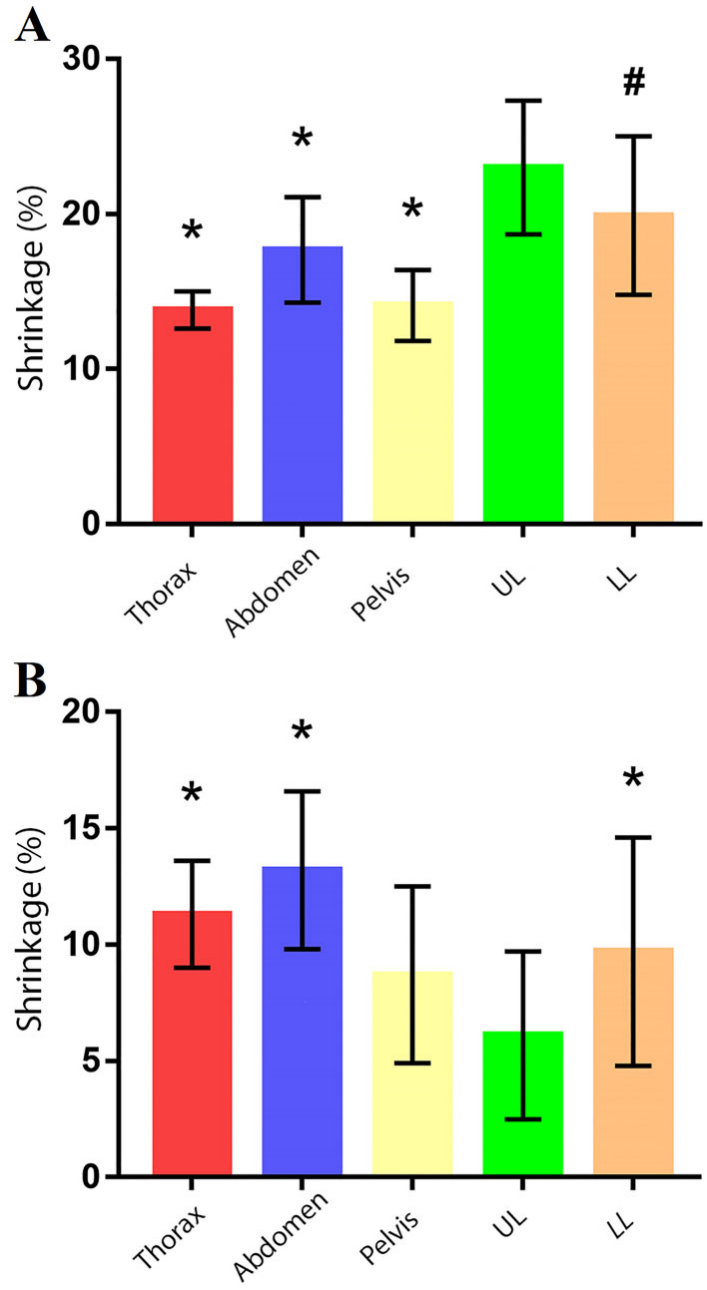
Mean (SD) percent volumetric shrinkage of the anatomical segments by silicone tested: S10 (**A**) and P1 (**B**). *P<0.05 vs UL; ^#^P<0.05 vs thorax (ANOVA). UL: upper limb; LL: lower limb.

**Figure 6:**
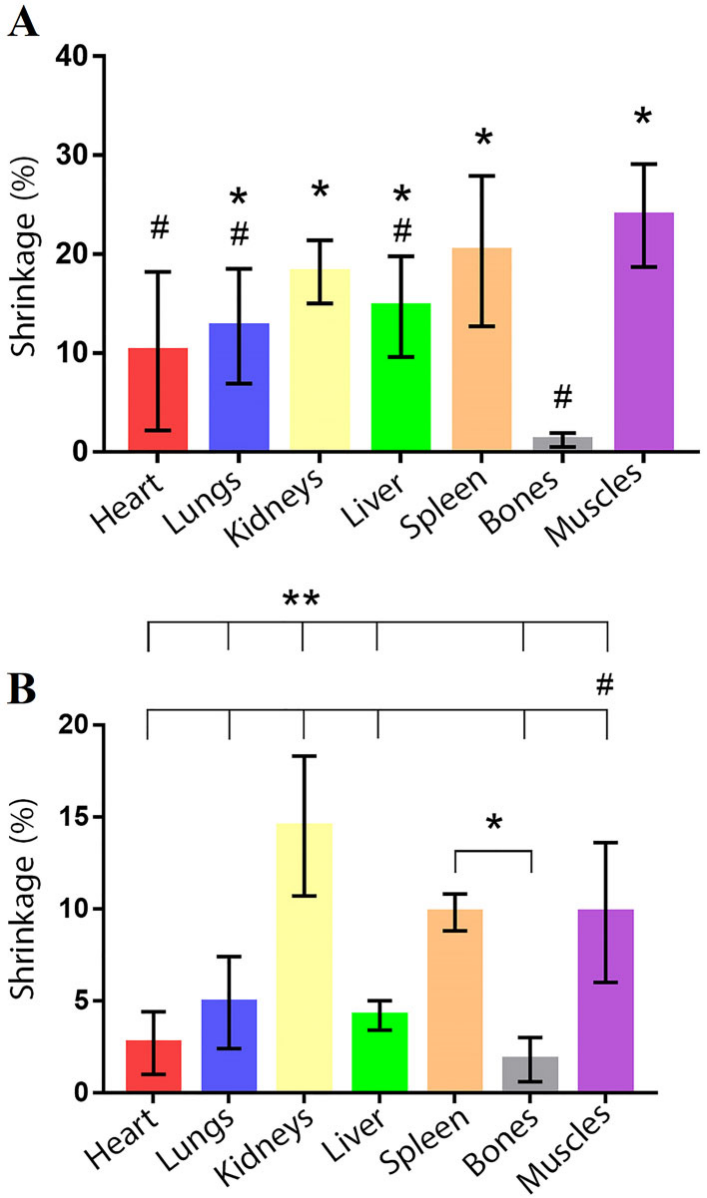
Mean (SD) percent shrinkage of the anatomical segments by silicone tested: S10 (**A**) and P1 (**B**). *P<0.05 *vs* bones; **P<0.05 *vs* kidneys; ^#^P<0.05 *vs* muscles (ANOVA).

To compare the means of volumetric or superficial shrinkage of the different anatomical segments and tissues within the same experimental group (P1 and S10), a one-way ANOVA was performed (Figures 5 and 6). This comparison was made in pairs of each specimen.

The anatomical segments had relatively little variation of volumetric shrinkage within both groups of silicones. The segment with the highest shrinkage difference was the upper limb (UL), both for S10 and P1. In the case of S10, except for the LL (P=0.16), the UL showed significantly higher shrinkage for all other tested segments (P<0.05). For P1, the UL shrunk significantly less than the other groups (P<0.05), except the pelvis (P=0.87).

The different biological tissues tested also showed relatively little difference within the same silicone group. As expected, bones showed very low shrinkage, differing from several other tissues impregnated with both S10 and P1. With S10, the bones showed a difference from all other tissues, except the heart (P=0.09). With P1, bones had no significant difference with the heart (P=0.99), lung (P=0.05), and liver (P=0.75), whereas bone shrinkage was significantly different than the kidney, spleen, and muscle. The non-significant differences with bones are probably due to the high standard deviation and the small shrinkage caused by P1 in the mentioned tissues.

Muscle tissue presented a significant difference in shrinkage compared with other tissues, except for spleen with P=0.82 (S10) and P>0.99 (P1), and kidney with P=0.06 (S10).

Five of the seven (71%) shrinkage comparisons between the same types of tissues for the two silicones showed a significant difference. The comparison between different types of tissue within the same silicone group showed that 10/21 (48%) for P1 and 8/21 (38%) for S10, or 18/42 (43%) for both silicones, showed a significant difference. Therefore, the "silicone type" factor was more relevant than the "tissue type" factor for tissue shrinkage caused by the forced impregnation process.

Although the chemical curing stage causes a reduction in polymer volume, especially in polymers with lower molar mass, the shrinkage value reported by the manufacturers in the technical data sheets of the two silicones was <0.5%. This shrinkage value became insignificant compared to that caused by the plastination process itself, especially in the forced impregnation stage.

Plastination is a relatively complex tissue preservation technique with several steps to be followed, so it is very difficult to control all the factors influencing shrinkage (5,9,10). Small changes in the technique, such as differences in time and formaldehyde concentration in the fixation step, acetone concentrations used and number of baths in dehydration, rate and constancy in forced impregnation, and drainage and pre-curing times, can influence the retraction level. However, this study had rigorous standardization of samples and steps, submitting all analyzed slices to the same conditions.

There was no visual change of colors in the pieces with the plastination process of the two experimental groups (silicones).

Some limitations of this research can be mentioned: 1) there was no strict control of the fixation of the cadaver with formalin, as the research design took place after this stage. However, this can be useful for laboratories that intend to plastinate pre-existing anatomical collections; 2) there was no control of the volume of drained silicone before the curing step; 3) the small number of samples for some types of biological tissues; 4) the shrinkage of slices was measured after curing and, therefore, there may have been minimal influences from the polymerization process (maximum retraction of 0.5% at this stage) and; 5) nervous tissue retraction was not evaluated.

**Figure 7:**
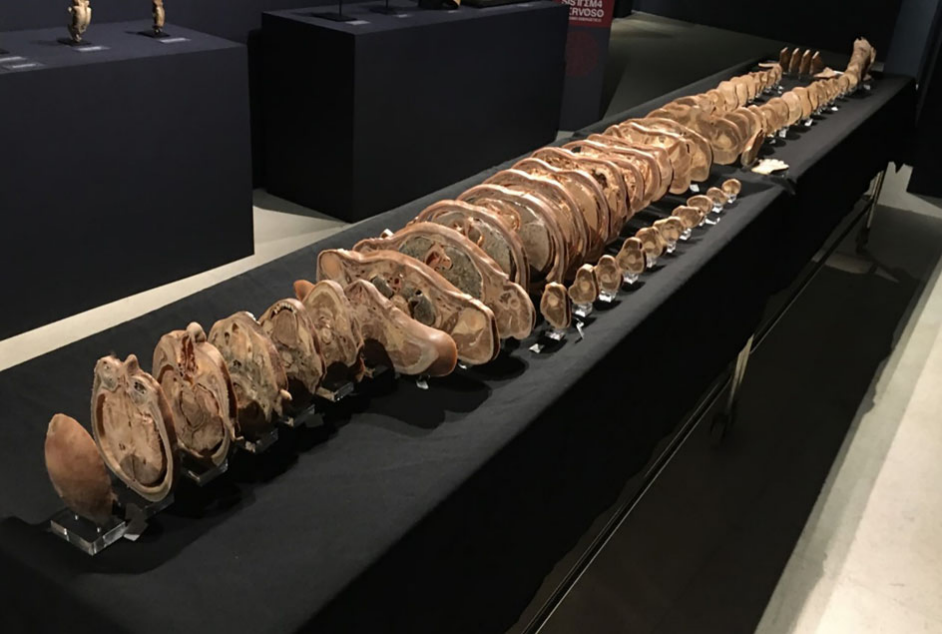
Plastinated specimen "tomography" on display at the Museum of Life Sciences (UFES).

After plastination and data collection for this study, part of the slices produced - the even-numbered ones - were allocated to the collection of the Anatomy Sector of the Department of Morphology of the UFES to be used in practical classes of health courses, and the other part - the odd-numbered pieces - was put on display at the Museum of Life Sciences (MCV - *Museu de Ciências da Vida*) of the UFES (Figure 7).

The production of specimens with less shrinkage, that is, closer to the actual size, are better for use in teaching and research in the health field. In clinical practice, an optimal plastination technique can be used to demonstrate diagnostic and therapeutic aspects of advanced surgical anatomy with specimens. Studies of morphometry and 3D reconstruction, tools widely used in clinical and applied anatomy research, may yield more accurate results. Plastination is also very useful for microscopic studies, such as histology and pathology. As concluded by Ramos et al. (17), specimens or fragments of plastinated biological tissues can be used in histological preparations to produce slides for electron and optical microscopy, for which specimens with less shrinkage are preferable. With this, tissues can be preserved almost indefinitely in a form that is easily stored, while maintaining the full potential for histological examination (11). More recent research has also shown the possibility of extracting intact genetic material from laminated tissues, including for PCR (polymerase chain reaction) application. For this, small modifications in the original technique were made, preserving intact DNA and facilitating its extraction. This discovery opens up many possibilities in the areas of basic and clinical sciences, epidemiology, forensic sciences, and legal medicine, since plastinated samples are extremely durable (they do not require maintenance) and are inert (18).

In conclusion, the P1 silicone caused a lower or equivalent tissue shrinkage, both in volume and area in all anatomical segments and different tissues analyzed compared to the S10 silicone. Subgroups that did not show a significant difference in shrinkage showed a tendency for lower shrinkage for the P1 silicone. The P1 silicone can therefore be used as an alternative to the S10 silicone, producing less tissue shrinkage and no difference in color and physical appearance.

## References

[1] von Hagens G, Tiedemann K, Kriz W (1987). The current potential of plastination. Anat Embryol (Berl).

[2] Latorre R, Henry R, Adds PJ, Baptista C (2019). The International Society for Plastination. Anat Histol Embryol.

[3] Sora MC, Latorre R, Baptista C, López-Albors O (2019). Plastination-a scientific method for teaching and
research. Anat Histol Embryol.

[4] Sora MC, Boia M, Banciu CD (2015). Silicone (BIODUR) Viscosity and Impregnation in
Plastination. Material Plastice.

[5] Starchik D, Henry RW (2015). Comparison of cold and room temperature silicone plastination
techniques using tissue core samples and a variety of
plastinates. J Plastinat.

[6] Sora MC, Cook P (2007). Epoxy plastination of biological tissue: E12
technique. J Int Soc Plastinat.

[7] Steinke H, Pfeiffer S, Spanel-Borowski K (2002). A new plastination technique for head slices containing
brain. Ann Anat.

[8] de Jong K, Henry RW (2007). Silicone plastination of biological tissue: cold-temperature
technique Biodur S10/S15 technique and products. J Int Soc Plastinat.

[9] Brown MA, Reed RB, Henry RW (2002). Effects of dehydration mediums and temperature on total
dehydration time and tissue shrinkage. J Int Soc Plastinat.

[10] Pereira-Sampaio MA, Marques-Sampaio BPS, Sampaio FJB, Henry RW (2011). Shrinkage of renal tissue after impregnation via the cold Biodur
plastination technique. Anat Rec (Hoboken).

[11] Judas F, Palma P, Falacho RI, Figueiredo H (2012). Estrutura e dinâmica do tecido ósseo.

[12] Monteiro YF, Juvenato LS, Bittencourt APSV, Siqueira BMM, Monteiro FC, Baptista CAC (2018). Influence of The temperature on the viscosity of different types
of silicone. J Plastinat.

[13] Junqueira LC, Carneiro J (2013). Histologia básica.

[14] Guimarães CF, Gasperini L, Marques AP, Reis RL (2020). The stiffness of living tissues and its implications for tissue
engineering. Nat Rev Mat.

[15] Thavarajah R, Mudimbaimannar VK, Elizabeth J, Rao UK, Ranganathan K (2012). Chemical and physical basics of routine formaldehyde
fixation. J Oral Maxillofac Pathol.

[16] Sora MC, Brugger PC, Strobl B (2002). Shrinkage during E12 plastination. J Int Soc Plastinat.

[17] Ramos ML, de Paula TAR, Zerlotini MF, Silva VHD, Carazo LB, de Paula MF (2018). A comparison of different de-plastination methodologies for
preparing histological sections of material plastinated with
Biodur^®^ S10/S3. J Plastination.

[18] Ottone NE, Baptista CAC, Del Sol M, Ortega MM (2020). Extraction of DNA from plastinated tissues. Forensic Sci Int.

